# Hepatocyte high-mobility group box 1 protects against steatosis and cellular stress during high fat diet feeding

**DOI:** 10.1186/s10020-020-00227-6

**Published:** 2020-11-25

**Authors:** Minjie Lin, Jungke Long, Wenbo Li, Chenxuan Yang, Patricia Loughran, Robert O’Doherty, Timothy R. Billiar, Meihong Deng, Melanie J. Scott

**Affiliations:** 1 Clinical Skills Training Center, The Second Xiangya Hospital of Central South University, Changsha, 410011 Hunan China; 2 Department of Surgery, University of Pittsburgh, Pittsburgh, PA 15213 USA; 3 Department of Cardiology, The Second Xiangya Hospital of Central South University, Changsha, 410011 Hunan China; 4 Department of Plastic Surgery, The Second Xiangya Hospital of Central South University, Changsha, 410011 Hunan China; 5 Tsinghua University School of Medicine, Beijing, 100084 China; 6 Center for Biologic Imaging, University of Pittsburgh, Pittsburgh, PA 15213 USA; 7 The Center for Metabolism and Mitochondrial Medicine of University of Pittsburgh, Pittsburgh, PA 15260 USA; 8 Pittsburgh Liver Research Center, University of Pittsburgh, Pittsburgh, PA 15213 USA; 9 University of Pittsburgh, NW653 MUH, 3459 Fifth Ave, Pittsburgh, PA 15213 USA; 10 University of Pittsburgh, NW607 MUH, 3459 Fifth Ave, Pittsburgh, PA 15213 USA

**Keywords:** NAFLD, Hepatic steatosis, Liver injury, ER stress, β-Oxidation

## Abstract

**Background:**

Circulating high-mobility group box 1 (HMGB1) plays important roles in the pathogenesis of nonalcoholic fatty liver disease (NAFLD). Intracellular HMGB1 is critical for the biology of hepatocytes. However, the intracellular role of HMGB1 in hepatocellular steatosis is unknown. Therefore, we aimed to investigate the role of hepatocyte-specific HMGB1 (HC-HMGB1) in development of hepatic steatosis.

**Methods:**

Wild type (WT) C57BL/6 and HC-HMGB1^−/−^ mice were fed high-fat diet (HFD) or low-fat diet (LFD) for up to 16 weeks.

**Results:**

As expected, HMGB1 translocated from nuclear into cytoplasm and released into circulation after HFD treatment. HC-HMGB1 deficiency significantly reduced circulating HMGB1, suggesting that hepatocyte is a major source of circulating HMGB1 during NAFLD. Unexpectedly, HC-HMGB1 deficiency promoted rapid weight gain with enhanced hepatic fat deposition compared with WT at as early as 4 weeks after HFD treatment. Furthermore, there was no difference between WT and HC-HMGB1^−/−^ mice in glucose tolerance, energy expenditure, liver damage or systemic inflammation. Interestingly, hepatic gene expression related to free fatty acid (FFA) β-oxidation was significantly down-regulated in HC-HMGB1^−/−^ mice compared with WT, and endoplasmic reticulum (ER) stress markers were significantly higher in livers of HC-HMGB1^−/−^ mice. In vitro experiments using primary mouse hepatocytes showed absence of HMGB1 increased FFA-induced intracellular lipid accumulation, accompanied by increased ER-stress, significant downregulation of FFA β-oxidation, and reduced oxidative phosphorylation.

**Conclusions:**

Our findings suggest that hepatocyte HMGB1 protects against dysregulated lipid metabolism via maintenance of β-oxidation and prevention of ER stress. This represents a novel mechanism for HMGB1-regulation of hepatocellular steatosis, and suggests that stabilizing HMGB1 in hepatocytes may be effective strategies for prevention and treatment of NAFLD.

## Background

The global epidemic of nonalcoholic fatty liver disease (NAFLD) poses a drastic threat to public health systems because of its increasing incidence around the world, especially in Western countries. In the United States, NAFLD is one of the most common chronic liver conditions, affecting an estimated 80–100 million individuals (Perumpail et al. 2017).The condition covers a wide range of liver changes, from simple steatosis and non-alcoholic steatohepatitis (NASH) to liver cirrhosis and hepatocellular carcinoma (Chen et al. 2013). The hallmark of NAFLD is abnormal lipid accumulation in the liver. Patients with NAFLD are at increased risk of liver-related as well as cardiovascular mortality, and NAFLD is closely linked to metabolic comorbidities, including obesity, insulin resistance, type 2 diabetes and metabolic syndrome (Calzadilla Bertot and Adams 2016). Although much attention has focused on NAFLD in recent decades, its pathogenesis remains largely obscure.

High-mobility group box 1 (HMGB1) is a highly conserved, ubiquitous nuclear protein constitutively expressed in nearly all cell types (Klune et al. 2008). Absence of HMGB1 in global knockout mice is lethal within a few days of birth, with newborn knockout mice succumbing to hypoglycemia (Calogero et al. 1999), although HMGB1 can be inducibly knocked out globally in adult mice (Aneja et al. 2019), suggesting a specific role for HMGB1 in development and early metabolism. HMGB1 has separate intracellular and extracellular functions (Deng et al. 2019). Under physiologic conditions, as a structural component in the chromatin complex it is involved in maintaining DNA structure in the nucleus and influences multiple processes such as DNA binding, replication, repair and bending, and gene transcription and stability (Abdulahad et al. 2010; Stros 2010; Stros et al. 2007). In response to cellular stress HMGB1 is mobilized from the nucleus into the cytoplasm, where it can influence intracellular processes such as autophagy. HMGB1 can also be actively and passively released from cells where it can act as a pro-inflammatory damage-associated molecular pattern (DAMP) (Jingjing et al. 2015).

Recently, HMGB1 has been identified as a potent inflammatory mediator in several liver diseases (Chen et al. 2013), and mounting evidence indicates an important role of extracellular HMGB1 in the development of NAFLD (Gaskell et al. 2018). HMGB1 translocates from nucleus to cytoplasm in NAFLD (Li et al. 2011), and circulating HMGB1 accelerates HFD-induced liver damage and inflammation during early stages of NAFLD (Li et al. 2011). In addition, circulating HMGB1 level also correlates with the severity of inflammation and hepatic fibrosis associated with NAFLD (Alisi et al. 2014; Ganz et al. 2015), and inhibition of HMGB1 release from hepatocytes, or treatment with neutralizing antibodies to HMGB1, attenuates liver damage during NAFLD (Zeng et al. 2015). However, intracellular functions of HMGB1 in the pathogenesis of NAFLD are unexplored. We and others have observed that total HMGB1 in hepatocytes decreases in response to fatty acid stimulation (Chen et al. 2018). However, whether loss of intracellular HMGB1 in hepatocytes also plays a role in the pathogenesis of NAFLD is unclear.

We explored roles of hepatocyte-specific HMGB1 (HC-HMGB1) using cell-type specific knockout mice, generated using cre-lox technology (Huang et al. 2014), to mimic in vivo the loss of intracellular HMGB1. We found that HC-HMGB1^−/−^ mice had early, rapid weight gain with significantly increased lipid accumulation compared with WT controls. Liver and hepatocytes from HC-HMGB1^−/−^ mice also had increased evidence of endoplasmic reticulum (ER) stress, which was associated with decreased hepatic β-oxidation. Our findings uncover a novel role for intracellular HMGB1 in hepatocytes in the regulation of lipid metabolism.

## Methods

### Animal use and treatment

All animal protocols were approved by the animal care and use committee of the University of Pittsburgh, and experiments were performed in strict adherence to the National Institutes of Health Guidelines for the Use of Laboratory Animals. Male wild-type (WT) C57BL/6 mice were purchased from The Jackson Laboratory (no.000664). HC-HMGB1^−/−^ mice were bred at our facility as described previously (Huang et al. 2014). Mice were housed four per cage. Starting at 10 weeks of age, mice of similar starting weights were randomized to either get high-fat diet (HFD) (45%kcal%fat; D12451, Research Diets, New Brunswick, NJ, USA) or low-fat diet (LFD) (10%kcal%fat; D12450K, Research Diets, New Brunswick, NJ, USA) for up to 16 weeks. See Additional file 1: Table S1 for Composition of rodent special diets. Mouse weight and food intake were measured weekly. Housing conditions and access to food and water were the same for all mice. Mice were euthanized under ether anesthesia and blood was obtained by cardiac puncture. Liver tissue was removed after perfusion with cold phosphate-buffered saline, and then either immediately fixed in 2% paraformaldehyde or snap-frozen in liquid nitrogen.

### Body fat composition, energy expenditure and glucose tolerance test (GTT)

Whole-body fat was measured in conscious mice using magnetic resonance spectroscopy (EchoMRI-100; Echomedical Systems, Houston, TX) (Chartoumpekis et al. 2015). To evaluate energy expenditure, O_2_ consumption was monitored by The Comprehensive Lab Animal Monitoring System (CLAMS) (Columbus Instruments, Columbus, OH). Blood glucose was measured using a TRUEtrack blood glucose meter (TRIVIDIA, Fort Lauderdale, FL, USA) in blood collected from the tails of mice. The TRUEtrack® System exceeds the minimum International Organization for Standardization (ISO) standards for accuracy with 96.5% of results within the ISO defined limits. Mice were fasted for 6 h before the test with free access to water. Blood glucose was then measured just before the intraperitoneal glucose injection (1 g/kg body wt in saline) and subsequently at 15, 30, 60, 90, and 120 min post-administration.

### Biochemical analyses

Serum alanine amino-transferase (ALT) levels were measured using the DRI-CHEM 4000 Chemistry Analyzer System (Heska, Des Moines, IA). Serum interleukin (IL)-6 levels in mice were detected by an enzyme-linked immunosorbent assay (ELISA) kit (R&D Systems, no.M6000B; sensitivity: 1.8 pg/mL). HMGB1 was quantified using by ELISA (TECAN, no.ST51011; sensitivity: 2.5 ng/mL).

### Histology

Specimens were fixed in 10% neutral buffered formalin, paraffin embedded, and sectioned. Hepatic lipid accumulation was evaluated using hematoxylin and eosin (H&E)-stained histological sections. Steatosis was graded as: 0, 0–5% of the hepatocytes in the section are steatotic; 1, greater than 5–33% of hepatocytes are steatotic; 2, greater than 33–66%; and 3, greater than 66% (Ryu et al. 2015). All histology was assessed by an investigator blinded to treatment group.

### Immunofluorescent staining

Liver tissue was removed after perfusion with cold phosphate-buffered saline and 2% paraformaldehyde. Tissue was then placed in 2% paraformaldehyde for an additional 2 h of further fixation, followed by three changes in 30% sucrose in distilled water over 24 h. Tissue sections of 5 μm were incubated with 5% normal goat serum for 45 min. Samples were incubated with 2 μg/mL anti-HMGB1 antibody (rabbit IgG, Abcam, ab18256) for 1 h. Sections were then incubated with Alexa 488-conjugated F-actin phalloidin (1:500, Invitrogen, San Diego, CA, USA); Cy3-conjugated goat anti-rabbit IgG (1:1000, for anti-HMGB1 antibody, Jackson Immunoresearch, no.111165003) for 1 h. A Hoechst nuclear stain was applied for 30 s and slides were prepared for imaging. Imaging conditions were maintained at identical settings within each antibody-labeling experiment with original gating performed using the negative control. Large area images in X and Y using a Nikon A1 confocal microscope (purchased with 1S10OD019973-01 awarded to Dr. Simon C. Watkins).

### Mouse hepatocyte isolation and cell culture

Primary mouse hepatocytes from WT and HC-HMGB1^−/−^ mice were isolated and plated as previously described (Scott et al. 2009). Hepatocytes (150,000 cells/mL) were plated on gelatin-coated culture plates or coverslips precoated with Collagen I (Thermo Fisher Scientific, no.08774383) in Williams E medium with 10% calf serum, 15 mM HEPES, 10^−6^ M insulin, 2 mM l-glutamine, 100 U/mL penicillin, and 100 U/mL streptomycin. Palmitic acid (PA; Sigma-Aldrich, no.P0500)-induced fat accumulation in vitro in hepatocytes was established as previously described (Li et al. 2011; Zeng et al. 2015; Malhi et al. 2006). PA was dissolved in 95% ethanol at 100 mM stock solution, which was then mixed with Williams medium E containing 10% calf serum to 8 mM stock concentration. PA concentration used ranged from 200 to 800 μM, which is similar to fasting plasma total free fatty acid (FFA) concentration in human nonalcoholic steatohepatitis (Takahara et al. 2017). Hepatocytes were allowed to attach to plates for 6 h and cultured in serum-free media for 12 h prior to the PA treatment. Isolated hepatocytes were exposed to tunicamycin (TM; Sigma-Aldrich, no.654380) (2 μg/mL for 6 h) to induce ER stress, or pretreated with 4-phenylbutyric acid (PBA; Sigma-Aldrich, no.P21005) (200 μM for 12 h) to inhibit ER stress.

### Oxygen consumption rate (OCR) measurement

WT and HC-HMGB1^−/−^ mouse hepatocytes were plated in XF-96 cell culture plates (10^4^ cells/well; Agilent, no.101085) overnight. Hepatocytes were then washed and incubated for 1 h in XF assay medium (unbuffered DMEM pH 7.4) in a non-CO_2_ incubator at 37 °C as per manufacturer’s instructions (Agilent). Real time measurements of hepatocyte OCR were performed using an XF-96 Extracellular Flux Analyzer (Agilent). Three consecutive measurements were obtained under basal conditions and after the sequential addition of 4 μM oligomycin, to inhibit mitochondrial ATP synthase (complex V); 1 μM FCCP (fluoro-carbonyl cyanide phenylhydrazone), a protonophore that uncouples ATP synthesis from oxygen consumption by the electron-transport chain providing a maximal OCR value; and 1 µM rotenone which inhibits the electron transport chain providing a baseline/minimum value.

### Human hepatocytes isolation and culture

Human hepatocytes were isolated from histologically normal liver and were kindly provided by Dr. David Geller (University of Pittsburgh Department of Surgery, Pittsburgh, PA) according to a protocol approved by the Institutional Review Board (Nussler et al. 1995). Human hepatocytes were prepared by a three-step collagenase perfusion technique. Isolated human hepatocytes were cultured in William's medium E (Invitrogen, no.12551032) supplemented with 5% calf serum (GE Healthcare Life Sciences), penicillin (100 U/mL), streptomycin (100 U/mL), 2 mM l-glutamine, and 15 mM HEPES.

### LDH cytotoxicity assay

Culture medium from treated hepatocytes (50 μL) was transferred to a 96-well plate and the LDH reaction was performed using Pierce LDH Cytotoxicity Assay Kit (Thermo Fisher Scientific, Waltham, MA, USA) following manufacturer's instructions. Absorbance at 680 nm (background signal) was subtracted from the absorbance at 490 nm. LDH activity was normalized to protein concentration and results are shown as fold of controls. At least three independent experiments with three replicates each were performed.

### Oil red O staining

Hepatocytes from each experimental group plated in six-well plates were rinsed three times with PBS, fixed in 4% paraformaldehyde for 30 min, stained for 60 min at RT in freshly diluted Oil Red O solution (0.5% Oil Red O in isopropanol: H_2_O = 3:2), rinsed three times with PBS, redyed for 30 s in hematoxylin staining solution and rinsed with PBS twice. Intracellular lipid droplets were imaged with Nikon TS100 inverted microscope connected to a digital camera.

### Real-time polymerase chain reaction (RT-PCR) analyses

Total RNA was extracted from liver tissue or hepatocytes using RNeasy Mini Kit (Qiagen) according to the manufacturer's instructions. Total RNA (1 μg) was reverse transcribed using an iScript cDNA Synthesis Kit (Bio-Rad Laboratories, no. 1708891), according to the manufacturer's instructions. The samples were then diluted, and the same amount of cDNA was added to each reaction on each plate. The iTaq universal SYBR Green supermix (Bio-Rad Laboratories, no. 1725121) and a different primer were used for gene expression analyses. All samples were run in triplicate, and the experiment was repeated three times. Primers used for carnitine-palmitoyltransferase 1α (CPT-1α), medium-chain acyl-CoA dehydrogenase (MCAD), long-chain acyl-CoA dehydrogenase (LCAD), very-long chain acyl-CoA dehydrogenase (VLCAD), fatty acid synthase (FAS), lipoprotein lipase (LPL), sterol regulatory element-binding protein 1 (SREBP-1), peroxisome proliferator-activated receptor gamma (PPAR-γ), adipophilin, collagen type 1A1 (COL1A1), collagen type 1A2 (COL1A2), tissue inhibitors of metalloproteinases (TIMP), α-smooth muscle actin (α-SMA), stearoyl-CoA desaturase-1 (SCD-1), and β-actin were ordered from Qiagen (Hilden, Germany). See Additional file 1: Table S2 for PCR primer sequences. Thermal cycling conditions were 10 min at 95 °C, followed by 40 cycles of 95 °C for 15 s and 60 °C for 1 min on a sequence detection system (ABI PRISM 7000; Applied Biosystems). Each gene expression was normalized with β-actin mRNA content.

### Immunoblotting

Western blot was performed using whole-cell lysates from either liver tissue or hepatocytes, as previously described (Scott et al. 2009). Total protein concentration of 1 µg/µl was loaded into the 12% gel for each specific Western blot analysis. Membranes were incubated overnight with antibodies against Akt (Cell Signaling, no. 9272), phospho-Akt (p-Akt) (Ser473) (Cell Signaling, no. 9271), HMGB1 (Abcam, ab18256), β-actin (Abcam, ab8226), GAPDH (Abcam, ab8245), ATF6 (Abcam, ab37149) and CHOP (Abcam, ab11419). Secondary antibodies (no. 31460 and no. 31430) were from Thermo Fisher Scientific. For Western blot analyses, cell lysis buffer (1:10, Cell Signaling, no. 9803) was used for whole cell lysis and tissue lysis together with protease inhibitors. Western gel images were quantified by densitometry analyses using ImageJ software and presented as a ratio of loading controls.

### Statistical analyses

Data are presented as mean ± standard error of mean (SEM). Experimental results are analyzed for their significance by Student’s *t*-test. Significance was established at the 95% confidence level (P < 0.05).

## Results

### HFD triggers HMGB1 loss in hepatocytes and increases serum HMGB1 level

**Fig. 1:**
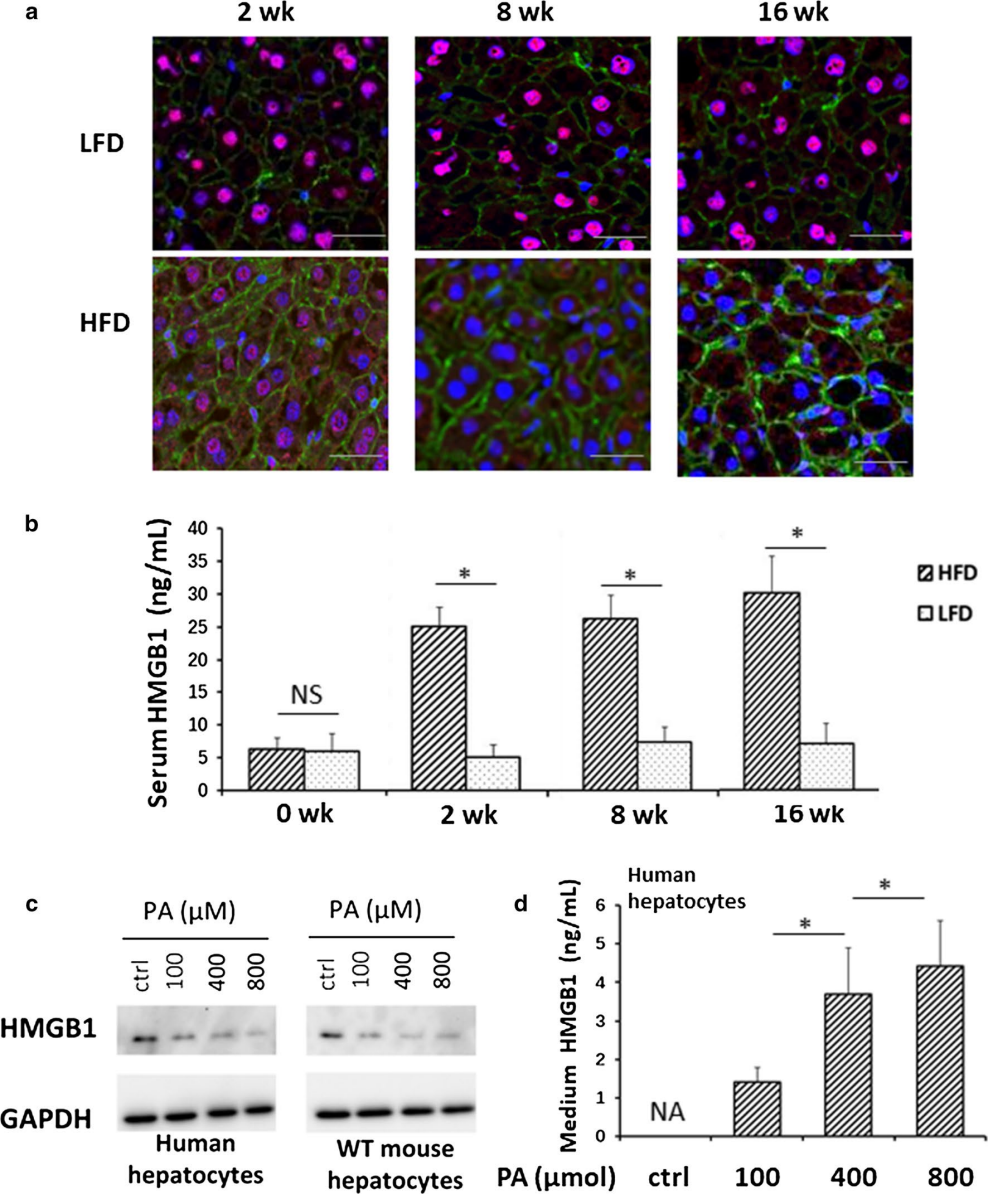
HMGB1 mobilization from hepatocytes of human and mice during HFD feeding. **a** Representative confocal immunofluorescence imaging of liver tissue of WT mice after 2, 8 and 16 weeks of LFD or HFD feeding. HMGB1 (red), actin (green) and 4′,6-diamidino-2-phenylindole (DAPI) nuclear staining (blue). Scale bars represent 20 μm. × 60 magnification. **b** Serum HMGB1 levels in WT mice after LFD or HFD feeding for 2, 8 and 16 weeks. N = 8/gp; data represent mean ± SEM; *P < 0.05 between indicated groups. **c** Representative Western blot images of HMGB1 in whole cell lysates of human (left) and WT mouse (right) hepatocytes at 24 h after up to 800 μM palmitic acid (PA) treatment. Images representative of at least three repeats. Ctrl = no treatment. GAPDH was used a loading control. **d** HMGB1 levels in the culture medium of human hepatocytes with PA exposure. Data presented as mean ± SEM. Data representative of at least three repeats. *P < 0.05 between indicated groups

Extracellular HMGB1 has been identified as a mediator of HFD-induced liver damage and inflammation during the early stages of NAFLD (Li et al. 2011). We examined HMGB1 expression, cellular location and release into circulation in HFD-fed mice. In WT mice as early as 2 weeks of HFD there was significant movement of HMGB1 into the cytoplasm and intercellular space of hepatocytes and this increased in a time-dependent manner. In contrast, HMGB1 remained in the nucleus of hepatocytes in the livers of LFD-treated WT mice (Fig. 1a). Furthermore, serum HMGB1 levels increased 5 folds in HFD treated mice compared with LFD-treated mice after only 2 weeks of HFD, and this was maintained until 16 weeks (Fig. 1b). Levels did not change over time in LFD treated WT mice. This confirms mobilization of HMGB1, and relative deficiency of HMGB1 in hepatocytes after HFD feeding. We also treated human and mouse hepatocytes with palmitic acid (PA), the most abundant fatty acid in HFD and in serum of NAFLD patients (Gambino et al. 2016), and assessed HMGB1 levels. In both human and mouse cells HMGB1 levels decreased and HMGB1 levels in the culture medium increased with PA exposure, suggesting similar mobilization (Fig. 1c, d).These results indicate that hepatocytes loss of HMGB1 and HC-HMGB1 is a major source of circulating HMGB1 during the development of NAFLD.

### HC-HMGB1 deficiency promotes early HFD-induced weight gain and obesity

**Fig. 2:**
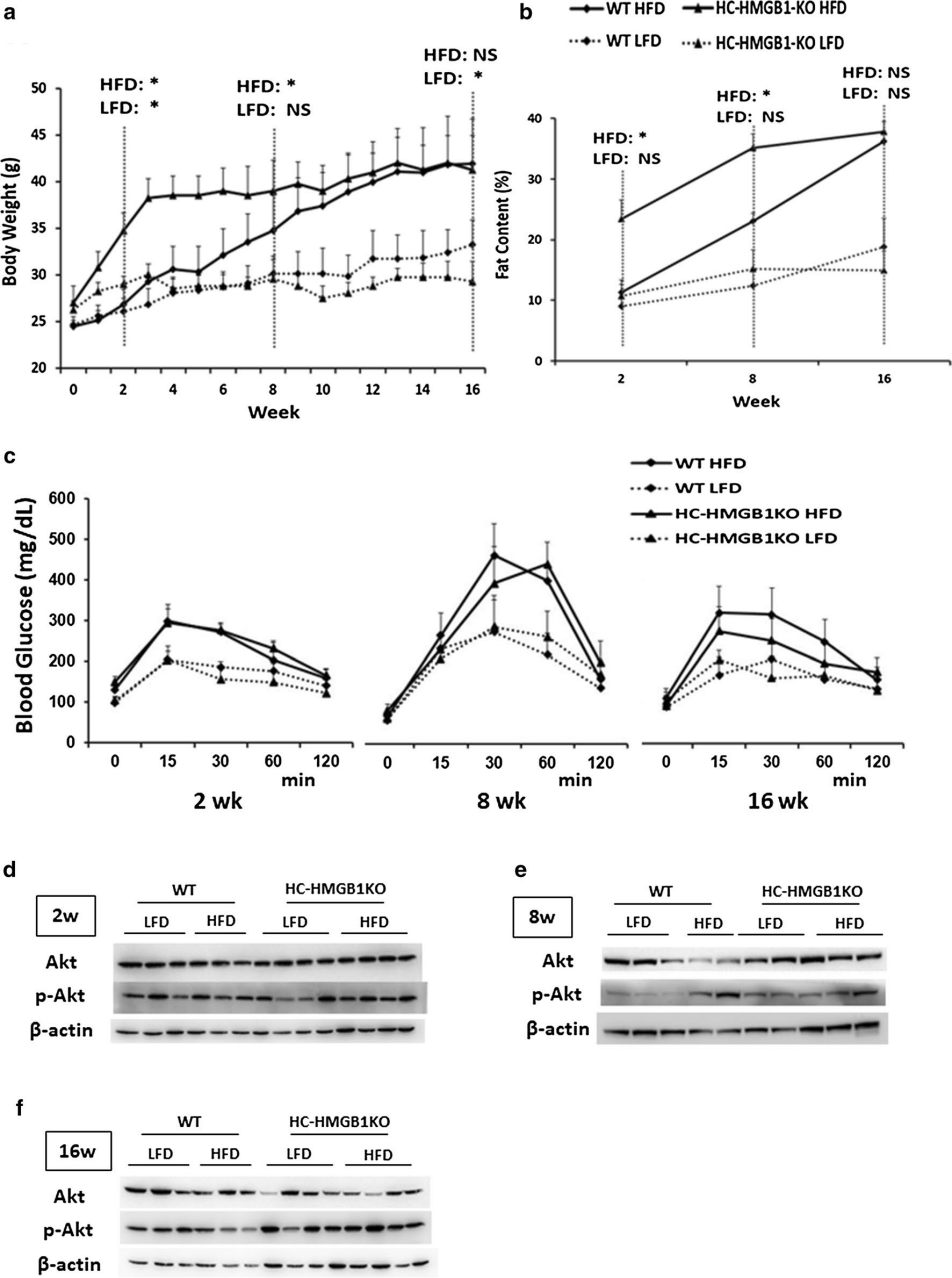
Body weight, fat content and glucose metabolism in WT and HC-HMGB1^−/−^ mice up to 16 weeks after LFD or HFD feeding. **a** Body weight measurements of WT and HC-HMGB1^−/−^ (KO) mice. **b** Fat content. **c** Blood glucose tolerance test. *P < 0.05 between WT LFD and KO LFD, or WT HFD and KO HFD groups; NS, not significant. **d**–**f** The insulin signaling. Western blot analyses of levels of Akt and p-Akt (insulin signaling markers) in WT and HC-HMGB1^−/−^ (KO) liver at **d** 2 weeks, **e** 8 weeks and **f** 16 weeks after LFD or HFD feeding. Lysates from one mouse liver per lane. Images representative of at least three repeated experiments

Since HMGB1 is closely linked to obesity and obesity-induced inflammation (Zhang et al. 2017), we monitored the weight changes of WT and HC-HMGB1^−/−^ mice weekly. The body weight increase of mice was greater in the HFD group than in the LFD group over the 16-week experiment (Fig. 2a). Interestingly, HC-HMGB1^−/−^ mice showed rapid, early weight gain, particularly over the first 4 weeks of HFD feeding, and gained significantly more body weight than WT mice by 8 weeks (Fig. 2a). However, body weight of the HC-HMGB1^−/−^ mice was plateaued after 8 weeks, and was similar to that of the WT mice by 16 weeks. The weight gain of HFD-fed HC-HMGB1^−/−^mice corresponded with increased body fat content (Fig. 2b), suggesting altered lipid metabolism or storage.

### HC-HMGB1 deletion has no effects on glucose intolerance, food intake and energy expenditure

Since obesity can lead to metabolic syndrome and hyperglycemia, we assessed glucose tolerance in both WT and HC-HMGB1^−/−^mice. Glucose intolerance (Fig. 2c) was increased after HFD in both WT and HC-HMGB1^−/−^ mice to a similar extent. The insulin signaling in hepatocytes from both genotypes was also analyzed by detecting phosphorylation of Akt, and we found that p-Akt was not affected by the knockout of HC-HMGB1 gene (Fig. 2d–f). Dietary intake was also similar between WT and HC-HMGB1^−/−^mice throughout 16 weeks of HFD or LFD administration (Additional file 2: Fig. S1A). We also assessed energy expenditure in both strains and found no significant differences in O_2_ consumption (V_O2_) between WT and HC-HMGB1^−/−^mice (Additional file 2: Fig. S1B). These findings suggest that HC-HMGB1 may influence lipid metabolism in liver, and subsequent fat storage, without affecting systemic energy and glucose homeostasis.

### Increased hepatic steatosis and liver injury are observed in HC-HMGB1^−/−^after HFD

**Fig. 3:**
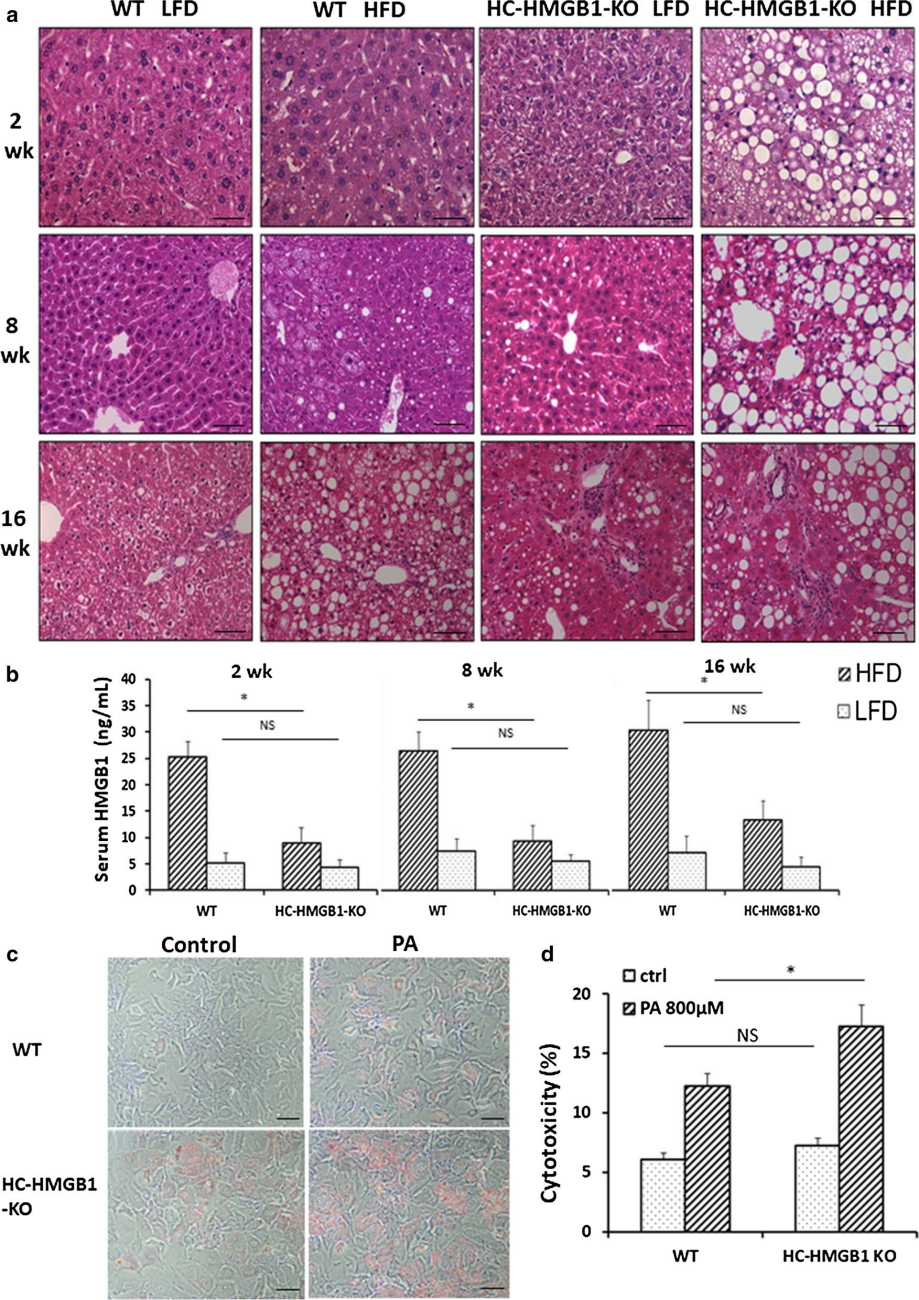
Steatosis, liver injury, inflammation and serum HMGB1 levels in WT and HC-HMGB1^−/−^ mice up to 16 weeks after LFD or HFD feeding. **a** Representative H&E stained liver sections. n = 4 for each group; × 40 magnification; scale bar, 50 μm. **b** Levels of serum HMGB1 in mice subjected to LFD or HFD for 2 weeks, 8 weeks and 16 weeks. **c** Lipid accumulation shown by Oil O Red Staining (red) in WT and HC-HMGB1^−/−^ mouse primary hepatocytes at 24 h after PA (800 μM) treatment. **d** Hepatocyte cytotoxicity (%) measured by LDH release in vitro after PA (800 μM) treatment for 24 h. Data show mean ± SEM for one experiment performed in triplicate and representative of three separate repeats. *P < 0.05 between indicated groups; *NS* not significant

H&E-stained liver sections were next assessed for hepatic steatosis. HC-HMGB1^−/−^ mice developed severe steatosis as early as 2 weeks after HFD, and this was maintained for up to 16 weeks (Fig. 3a).WT mice showed evidence of fat accumulation in liver after 16 weeks of HFD, but this was less extensive than in HC-HMGB1^−/−^ mice (Fig. 3a). Interestingly HC-HMGB1^−/−^ mice on LFD also showed increased fat accumulation by 16 weeks (Fig. 3a), which further suggests deficiencies in hepatocyte lipid metabolism and storage with HMGB1-deficiency. Hepatic steatosis score based on H&E-stained mouse liver sections is shown in Table 1. Consistently, hepatocytes lacking HMGB1 had increased lipid deposition at 24 h after PA treatment compared with controls, as shown by Oil O Red staining (Fig. 3c). Despite obvious increased hepatic steatosis in HC-HMGB1^−/−^mice early, serum levels of ALT (marker of liver injury) and IL-6 (systemic inflammation) were not significantly higher compared to controls mice until 16 weeks (Additional file 3: Fig. S2A, B). We also measured HMGB1 levels and found significantly lower levels of circulating HMGB1 in HC-HMGB1^−/−^ mice compared with WT (Fig. 3b), confirming the importance of liver and hepatocytes as sources of HMGB1 in the circulation. Given that circulating HMGB1 is a known inflammatory DAMP, it may be that the HC-HMGB1^−/−^ mice are relatively protected from systemic inflammation despite increased hepatocyte stress and damage from lipid accumulation. Indeed, using an in vitro assay we showed that HC-HMGB1^−/−^ hepatocytes were more susceptible to PA-induced cell death (Fig. 3d). These results indicate that loss of HMGB1 increases liver injury and cytotoxicity in hepatocytes during NAFLD.

**Table 1:** Hepatic steatosis score based on H&E-stained mouse liver sections

Group	Hepatic Steatosis Score			
	LFD		HFD	
	WT	HC-HMGB1^*−/−*^	WT	HC-HMGB1^*−/−*^
2 w	0 ± 0	0 ± 0	0 ± 0	2 ± 0
8 w	0 ± 0	1 ± 0	1 ± 0	3 ± 0
16 w	0 ± 0	1.75 ± 0.50	3 ± 0	3 ± 0

n = 4 for each group; data presented as mean ± SEM

### Loss of HC-HMGB1 induces ER stress

**Fig. 4:**
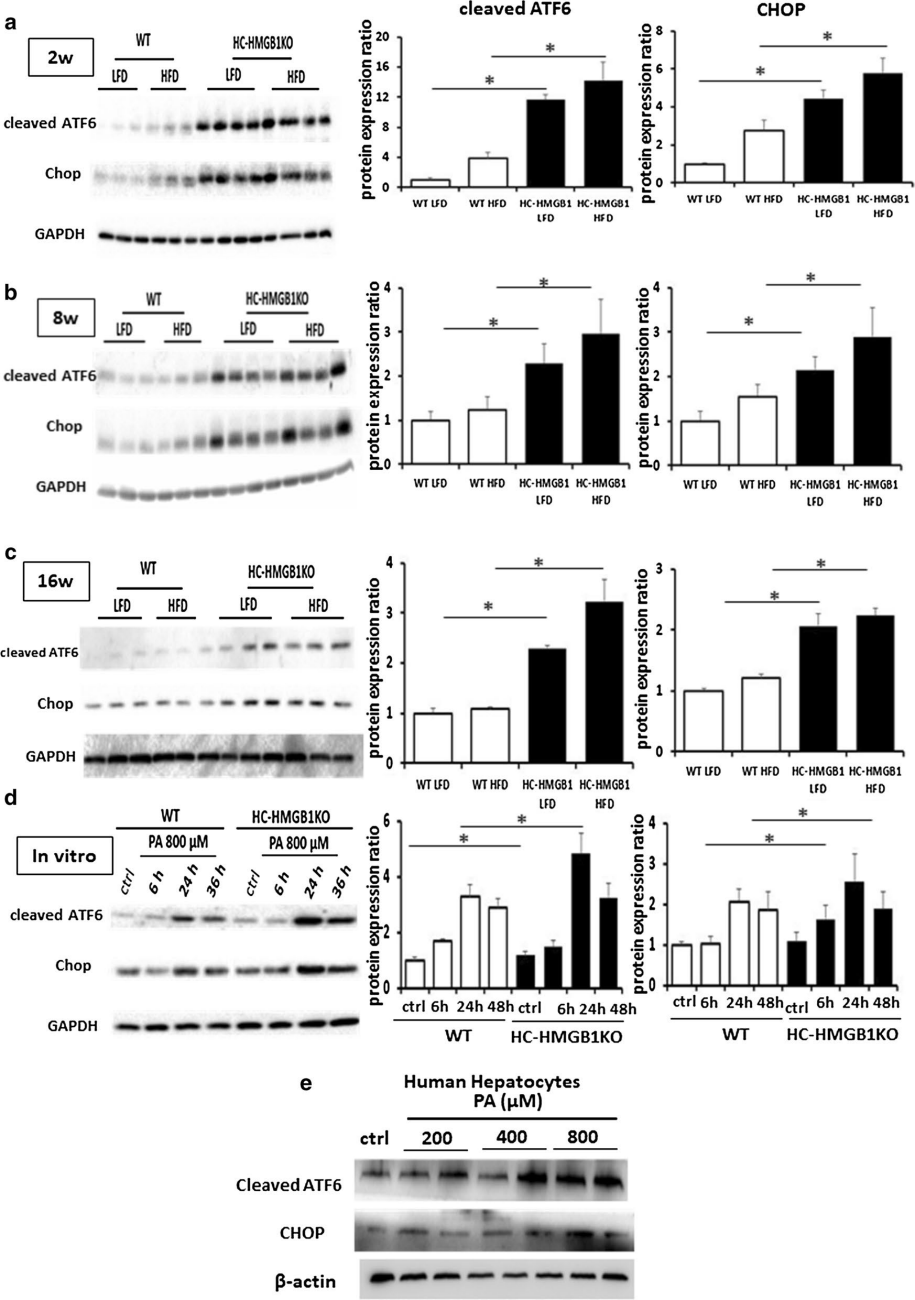
ER stress in WT and HC-HMGB1^−/−^ (KO) liver up to 16 weeks after LFD or HFD feeding, and in hepatocytes after PA treatment. Western blot analyses of levels of cleaved ATF6 and CHOP (ER stress markers) in WT and HC-HMGB1^−/−^ (KO) liver at **a** 2 weeks, **b** 8 weeks and **c** 16 weeks after LFD or HFD feeding. Lysates from one mouse liver per lane. **d** Western blot of WT and HC-HMGB1^−/−^ (KO) hepatocyte whole cell lysates in control (ctrl—no treatment), or time points up to 36 h after PA treatment. Gray analyses was performed for quantization of protein expression. Three independent experiments were performed. All data are means ± SEM. *P < 0.05. **e** Western blot of human hepatocyte whole cell lysates in control (ctrl—no treatment), or 24 h after PA treatment. GAPDH and β-actin were used as a loading control. Images representative of at least three repeated experiments

It is increasingly clear that perturbed ER proteostasis in hepatocytes is involved in pathogenesis of NAFLD through the activation of ER stress signaling (Pagliassotti 2012), and pharmacological induction of ER stress increases hepatic steatosis during NAFLD (Rutkowski et al. 2008; Lebeaupin et al. 2018). We therefore assessed ER stress in livers of WT and HC-HMGB1^−/−^ mice after LFD or HFD feeding. ER stress, indicated by levels of cleaved ATF6 and CHOP, was enhanced in HC-HMGB1^−/−^ mice compared with WT regardless of HFD or LFD feeding (Fig. 4a–c). Similarly, HMGB1^−/−^ hepatocytes treated with PA in vitro had increased cleaved ATF6 and CHOP compared to similarly treated WT cells (Fig. 4d). Consistently, the levels of cleaved ATF6 and CHOP was increased after PA treatment in human hepatocytes (Fig. 4e). These results suggest a role for HMGB1 in homeostasis in hepatocytes via regulation of ER stress.

### Deletion of HC-HMGB1 down-regulates hepatic gene expression related to β-oxidation

**Fig. 5:**
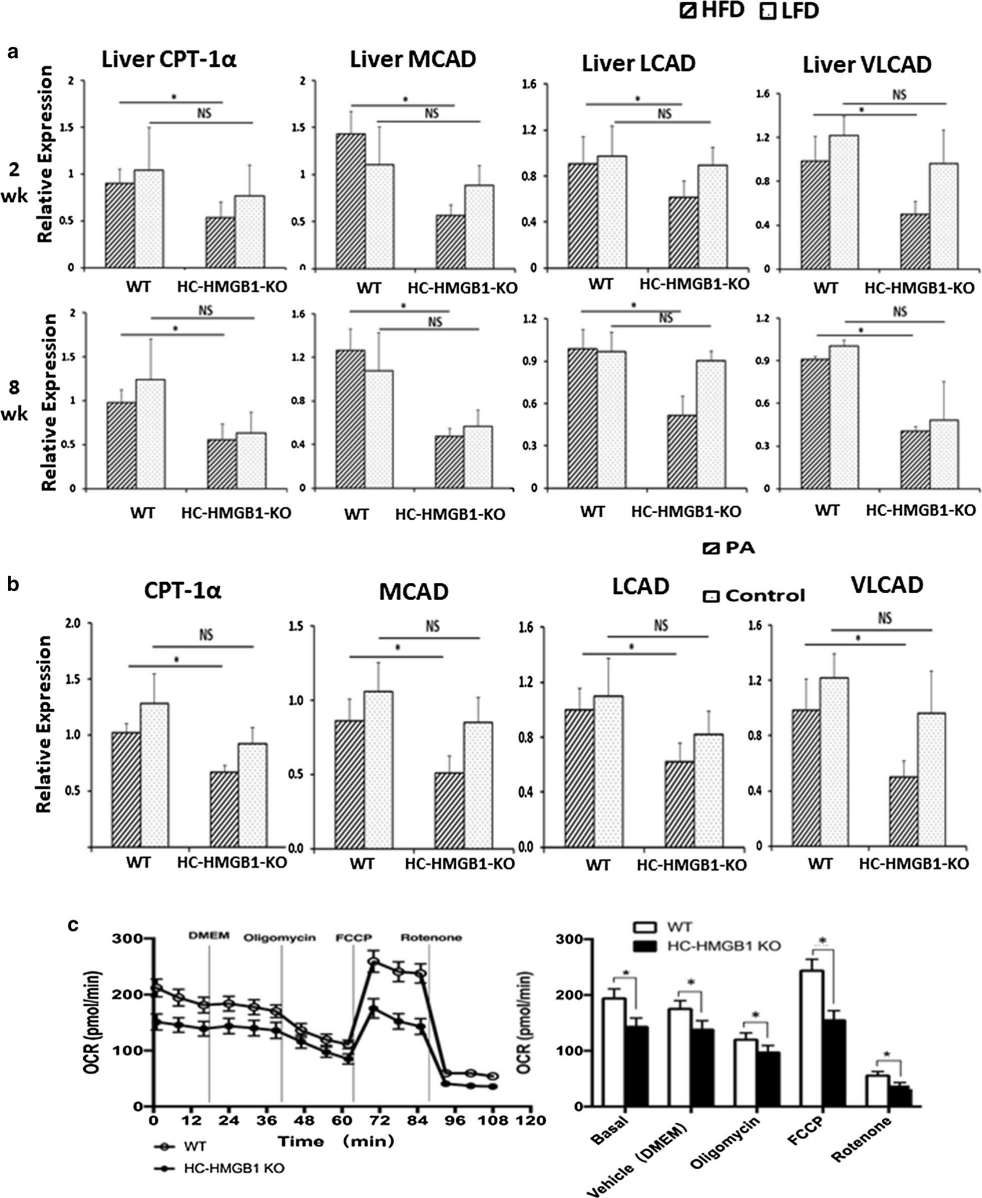
Deletion of HC-HMGB1 down-regulates hepatic gene expression related to β-oxidation in WT and HC-HMGB1^−/−^ (KO) mice up to 16 weeks and mouse hepatocytes at 24 h after PA treatment. **a** Relative gene expression of hepatic CPT-1, MCAD, LCAD and VLCAD in WT and HC-HMGB1-KO mice on LFD or HFD (n = 4 for each group). **b** Relative gene expression of CPT-1α, MCAD, LCAD and VLCAD in mouse hepatocytes at 24 h after PA treatment (800 µM). **c** OCR in WT and HC-HMGB1^−/−^ mouse hepatocytes. OCR tracing includes vertical lines indicating time of addition of mitochondrial inhibitors: oligomycin (4 µM), FCCP (1 µM), and rotenone (1 µM). OCR was normalized to protein content and is shown as fold changes of normoxic control. Data are mean ± SEM, graphs show collated data from 10 individual measurements from at least three separate experiments. *P < 0.05 between indicated groups; *NS* not significant

Given our data above suggesting a role for intracellular HMGB1 in lipid metabolism in hepatocytes, we measured mRNA levels of mouse liver enzymes involved in FFA β-oxidation, lipogenesis and lipolysis. Dysregulation of these pathways contributes to lipid metabolic disorders and lipid accumulation (Zechner et al. 2012; Bechmann et al. 2012; Ipsen et al. 2018). We screened multiple genes (Table 2), and found hepatic gene expression related to β-oxidation (CPT-1α, MCAD, LCAD and VLCAD) was significantly down-regulated in HC-HMGB1^−/−^ mice compared with WT throughout the 8 weeks of HFD feeding (Table 2 and Fig. 5a). To further investigate the underlying mechanisms we used our in vitro model of hepatocytes isolated from WT and HC-HMGB1^−/−^mice exposed to PA. Similar to our in vivo results, HMGB1^−/−^ hepatocytes also had significantly decreased CPT-1α, LCAD and VLCAD gene expression after stimulation with PA (Fig. 5b). Oxidative phosphorylation levels, and indication of metabolic activation of cells, were also lower in HC-HMGB1^−/−^ hepatocytes compared with WT (Fig. 5c). These data demonstrate that hepatocyte HMGB1 regulates lipid accumulation via β-oxidation-related gene expression, suggesting β-oxidation as a downstream target of intracellular HC-HMGB1 during NAFLD.

**Table 2:** Relative gene expression of hepatic CPT-1, MCAD, LCAD and VLCAD in WT and HC-HMGB1-KO mice on LFD or HFD for 8 weeks

Gene	mRNA relative expression					
	LFD			HFD		
	WT	HC-HMGB1^*−/−*^	P value	WT	HC-HMGB1^*−/−*^	P value
β-oxidation						
CPT-1α	1.24 ± 0.46	0.63 ± 0.24	0.069	0.98 ± 0.14	0.56 ± 0.18	**0.010**
MCAD	1.08 ± 0.35	0.57 ± 0.15	0.052	1.26 ± 0.20	0.48 ± 0.07	**0.002**
LCAD	0.97 ± 0.14	0.91 ± 0.07	0.450	0.99 ± 0.14	0.52 ± 0.14	**0.003**
VLCAD	1.01 ± 0.34	0.49 ± 0.27	0.054	0.91 ± 0.021	0.41 ± 0.0	** < 0.001**
Lipogenesis						
FAS	0.96 ± 0.05	0.87 ± 0.32	0.610	0.93 ± 0.41	0.78 ± 0.18	0.550
PPAR-γ	0.96 ± 0.04	0.70 ± 0.24	0.120	1.87 ± 0.66	1.69 ± 0.17	0.630
SCD-1	1.01 ± 0.29	1.47 ± 0.39	0.110	0.80 ± 0.10	2.32 ± 0.98	0.053
Adipophilin	0.89 ± 0.21	0.41 ± 0.38	0.091	2.78 ± 1.99	0.44 ± 0.49	0.096
Lipolysis						
LPL	1.18 ± 0.25	0.28 ± 0.30	0.004	0.16 ± 0.15	0.40 ± 0.17	0.079
SREBP1	1.42 ± 0.21	1.16 ± 0.21	0.130	0.96 ± 0.29	0.62 ± 0.14	0.098

n = 4 for each group; data presented as mean ± SEM

### ER stress is associated with lipid accumulation in hepatocytes

**Fig. 6:**
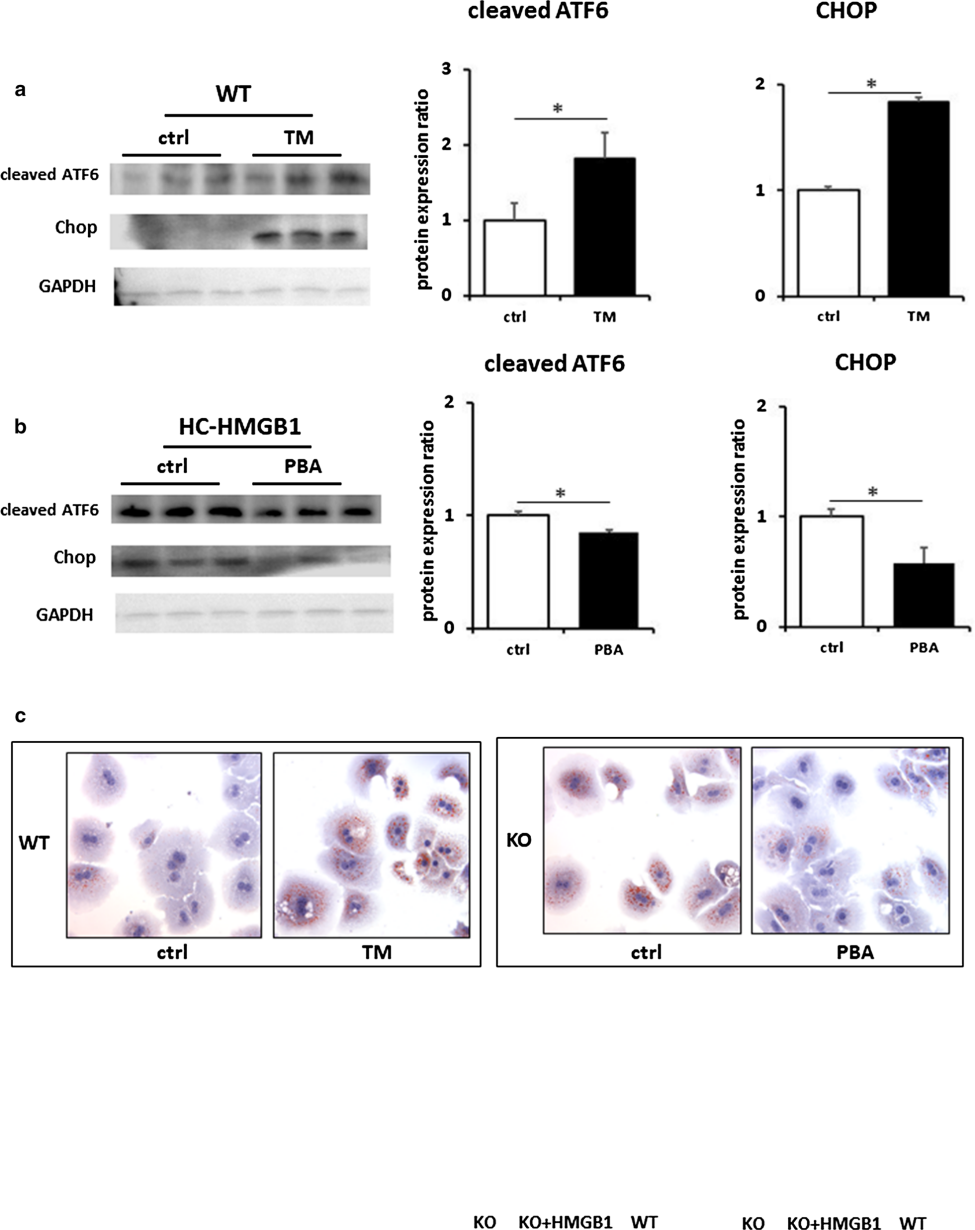

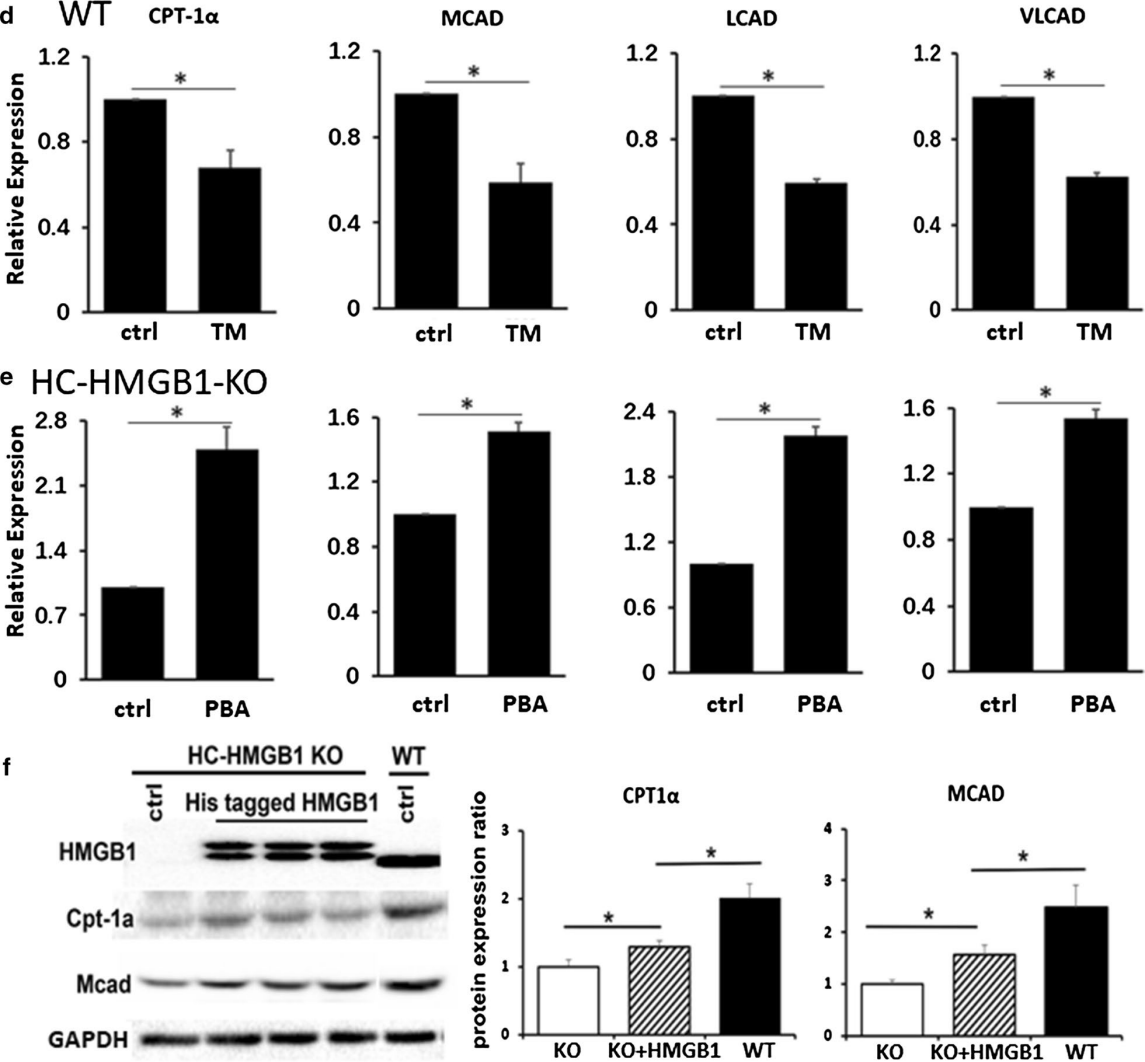
ER stress is associated with lipid accumulation in hepatocytes and suppression of β-oxidation gene expression. **a** Western blots showing levels of ER stress markers (cleaved ATF and CHOP) in hepatocytes. WT mice with and without treatment with ER stress inducer tunicamycin (TM; 2 μg/mL) for 6 h. GAPDH was used as a loading control. Images representative of at least three repeated experiments. Gray analyses was performed for quantization of protein expression. Three independent experiments were performed. All data are means ± SEM. *P < 0.05. **b** Western blots showing levels of ER stress markers (cleaved ATF and CHOP) in hepatocytes from HC-HMGB1-KO mice with and without treatment with ER stress inhibitor 4-phenylbutyric acid (PBA; 200 μM) for 12 h. **c** Lipid accumulation shown by Oil O Red Staining (red) in WT mouse hepatocytes treated with/without TM, or HC-HMGB1-KO mouse hepatocytes treated with/without PBA. x60 magnification. Representative images of multiple fields of view. Experiment repeated at least twice. **d** Relative gene expression of CPT-1α, MCAD, LCAD and VLCAD β-oxidation genes in WT mouse hepatocytes treated with/without TM; **e** Relative gene expression of CPT-1α, MCAD, LCAD and VLCAD β-oxidation genes in HC-HMGB1-KO mouse hepatocytes treated with/without PBA (n = 4 for each group). Data are expressed as mean ± SEM. *P < 0.05 between indicated groups. **f** Representative Western blot image of HMGB1, Cpt-1α and MCAD protein expression in whole cell lysates of WT and HC-HMGB1-KO hepatocytes at 40 h after transfection with control plasmid (ctrl) or plasmid expressing His-HMGB1. Quantification of relative protein expression shown below. Data presented as mean ± SEM. Data representative of at least three repeats. *P < 0.05 between indicated groups

Having demonstrated that steatosis in hepatocytes lacking HMGB1 is accompanied by ER stress, we wanted to examine this relationship further. We therefore induced ER stress in WT mouse hepatocytes, using ER stress inducer TM (2 μg/mL for 6 h). We confirmed induction of ER stress with TM (Fig. 6a) and saw enhanced intracellular lipid accumulation in TM-treated cells after PA treatment (Fig. 6c). Interestingly, we also saw suppression of the same β-oxidation genes (CPT-1α, MCAD, LCAD and VLCAD) in TM-treated WT hepatocytes suppressed in HMGB1^−/−^ liver and hepatocytes (Fig. 6d). We also inhibited ER stress in HMGB1^−/−^ hepatocytes using 4-phenylbutyrate (200 μM for 12 h), and confirmed that this was able to reduce ER stress in response to PA treatment (Fig. 6a, b). We saw corresponding reduced lipid deposition in cells (Fig. 6c), and increased expression of key β-oxidation genes (Fig. 6e). Next we assessed β-oxidation gene expression in WT and HC-HMGB1^−/−^ hepatocytes, as well as HC-HMGB1^−/−^ hepatocytes transfected with plasmids expressing His-tagged HMGB1. Induced HMGB1 expression in HC-HMGB1^−/−^ hepatocytes was able to significantly increase expression of CPT-1α and MCAD compared with control plasmid-treated cells (Fig. 6f). However, the levels were still significantly lower than in WT cells. These data suggest that ER stress and HMGB1 are intricately linked with hepatocyte lipid metabolism.

## Discussion

Understanding the mechanisms driving NAFLD and hepatosteatosis is key to developing new approaches to treatments. HMGB1 is a multifunctional protein that has specific biological functions dependent on its intracellular/extracellular location (Deng et al. 2019; Harris et al. 2012). Extracellular HMGB1, released either passively after lytic cell death or actively from stressed and damaged cells, has been shown to be an important mediator of liver injury in NAFLD (Li et al. 2011). It is also reported that HMGB1 mediates inflammatory pathway that is receptor for advanced glycation end products (RAGE) and redox signaling dependent and helps promote ectopic intestinal inflammation in NAFLD (Chandrashekaran et al. 2017).This is consistent with the role of HMGB1 as a damage-associated molecular pattern contributing to injury, inflammation, apoptosis, and immune responses (Chen et al. 2013).

Circulating HMGB1mainly comes from hepatocytes in multiple pathologies, including sepsis (Deng et al. 2018). Here we show that HC-HMGB1^−/−^ mice have significantly reduced circulating HMGB1 in response to HFD feeding (Fig. 3b), again confirming that hepatocytes are major sources of HMGB1. Recent studies suggest that HMGB1 is a key protein participating in the pathogenesis of acute liver injuries and chronic liver diseases (Chen et al. 2013; Gaskell et al. 2018) with circulating HMGB1 levels correlating with severity of inflammation and fibrosis in NAFLD (Alisi et al. 2014; Ganz et al. 2015). Increased circulating HMGB1 levels were confirmed in a mouse model of NAFLD, and neutralizing antibodies to HMGB1 were protective (Chen et al. 2018). Toll-like receptors (TLR)-2/4/9 and RAGE have all been shown to mediate HMGB1 signaling (Chen et al. 2013; Li et al. 2014). In 2011, Li et al. reported HMGB1 releasing from hepatocytes in response to FFA activates TLR4/MyD88 to induce cytokines expression in vitro and in vivo. Treatment with neutralizing antibody to HMGB1 protects against FFA-induced tumor necrosis factor alpha and interleukin-6 production (Li et al. 2011). Chandrashekaran et al. also identified systemic circulating HMGB1, acting via RAGE, as a key mediator accelerating intestinal inflammation in NAFLD (Chandrashekaran et al. 2017). Despite that all these published data indicated extracellular HMGB1 may be detrimental in liver damage, there still exists controversy over the role of circulating HMGB1 plays in liver injury and its association with serum ALT. Although HMGB1 is found to be correlated with ALT activity during clinical acetaminophen hepatotoxicity (Antoine et al. 2012), it is also reported that serum HMGB1 was not related to histological severity or serum ALT in adult and children NAFLD (Yates et al. 2017). In our mouse models, serum HMGB1 levels were significantly elevated by HFD from week 2, while ALT levels were not significantly different between HFD and LFD groups. To investigate the link among circulating HMGB1, serum ALT and liver damage, further studies to expand the results found here are warranted.

In sharp contrast to numerous studies examining the effect of extracellular HMGB1 on acute and chronic liver diseases, few studies have demonstrated the role of intracellular HMGB1 plays in liver damage, except that intracellular HMGB1 in hepatocytes has been shown to be protective during liver ischemia and reperfusion injury (Huang et al. 2014). So far, little is known about the role intracellular hepatocyte HMGB1 plays in the pathogenesis of NAFLD and liver cell injury. In this study, we uncover a novel pathway of hepatocyte HMGB1-mediated hepatoprotection from lipid accumulation, hepatosteatosis and liver damage during HFD feeding. Lack of HMGB1 increases ER stress in hepatocytes, and reduced β-oxidation resulting in increased lipid accumulation and cell injury.

ER plays an essential role in multiple cellular processes including secretory and transmembrane protein folding, calcium homeostasis and lipid biogenesis (Hetz 2012). Perturbation of ER homeostasis results in ER stress and activation of the unfolded protein response (UPR), which is initiated to restore ER proteostasis (Lodish et al. 2008), (Schröder and Kaufman 2005). Emerging evidence points to an important role of HMGB1 in UPR and ER stress induced by *Clostridium difficile* Toxin A, one of the major virulence factors of *C. difficile* infection (Liu et al. 2016). Exogenous recombinant HMGB1 also induced ER stress in CT26 cells (the murine colon adenocarcinoma cell line), while glycyrrhizin, an HMGB1 inhibitor, decreased ER stress (Liu et al. 2016). Again, extracellular HMGB1 seems to induce detrimental signaling and increases liver cell injury and stress. However, our data in this study show that intracellular HMGB1 can actually be protective and plays a role in maintaining ER homeostasis and preventing FFA-induced liver damage.

Maintenance of hepatic ER homeostasis is pivotal for sustaining membrane lipid composition and maintaining both intrahepatic and plasma lipid homeostasis (Ipsen et al. 2018). The hallmark of NAFLD is the abnormal lipid accumulation in the liver, which subsequently leads to cellular stress and hepatic injury. Excessive hepatic fat accumulation often coincides with perturbed ER homeostasis in hepatocytes (Lebeaupin et al. 2018), and ER stress has been associated with NAFLD pathogenesis (Pagliassotti 2012). ER membrane-localized transcription factors regulate de novo lipogenesis: sterol regulatory element-binding proteins (SREBP1c (*SREBF1*) for fatty acid synthesis and SREBP2 (*SREBF2*) for sterol synthesis (Ipsen et al. 2018). Hepatocytes also store lipids in the form of triglycerides that are synthesized from fatty acids and glycerol by ER-localized acyltransferase enzymes, including diacylglycerol acyltransferase (DGAT). Very-low-density lipoprotein (VLDL), which is assembled in the ER prior to trafficking to the Golgi (Reyes-Soffer et al. 2017), is an important mediator of lipid and cholesterol uptake into cells, including hepatocytes. Recently, Lebeaupin et al. reviewed available evidence linking ER stress to NAFLD and concluded that chronic ER stress can affect hepatic lipid metabolism directly by inducing de novo lipogenesis, and indirectly through the alteration of VLDL secretion, insulin signaling and autophagy (Lebeaupin et al. 2018). Conversely, increased hepatocyte lipid content can also initiate chronic ER stress (Lebeaupin et al. 2018), creating a potential feed-forward potentiation of NAFLD-mediated liver damage. Our data suggest intracellular HMGB1 is integral to these homeostatic pathways and contributes to maintaining lipid homeostasis under normal and pathogenic conditions.

Given all those published data on the detrimental effects of extracellular HMGB1, we were initially surprised by our findings that HC-HMGB1 is protective in HFD feeding. However, our subsequent findings suggest that the intracellular effects of HMGB1-deficiency on cell stress and injury outweigh any benefit of reduced circulating HMGB1, highlighting the importance of effects on cellular lipid metabolism in the pathogenesis of NAFLD.

In addition, in this study we identified a remarkable increase of body weight gain, fat content and hepatic lipid accumulation in HC-HMGB1^−/−^ mice at as early as 2 weeks after HFD administration. Blood glucose, food intake and energy expenditure did not significantly differ between HC-HMGB1^−/−^ and WT mice over the same period. It seems that HC-HMGB1^−/−^ mice were more susceptible to obesity and fatty liver when HFD-fed than WT mice at the early stage. Intracellular HMGB1 may associate with the susceptibility of body weight increase and hepatic lipid deposition. By administering anti-HMGB1antibody to C57Bl/6 mice, Montes et al. (Montes et al. 2015) previously reported that anti-HMGB1 treatment reduced weight gain in mice fed a high (60%)-fat diet from the 2nd week and thus HMGB1 was inferred to play a crucial role in weight gain at the early stage of obesity, though they did not observe differences in glucose, insulin tolerance and adipose tissue inflammation between HMGB1 neutralizing and control groups. Taking all of these evidences together, despite that the mechanism of HMGB1-mediated weight change is incompletely understood, we could draw a preliminary conclusion that HMGB1 may have a key role in weight change at the early stage of HFD. More experiments should be undertaken to elucidate the crosstalk among intracellular HMGB1 in hepatocytes, body weight and fat content.

In our study, it is also interesting to see increases in steatosis and ER stress without increases in serum IL-6 (systemic inflammation) or ALT (liver damage). Our interpretation is that endogenous hepatic HMGB1 is having multiple effects, and our data suggest that hepatocyte-derived HMGB1 not only regulates lipid metabolism within cells, but also plays a role in promoting systemic inflammation and augmenting hepatocyte cell death as part of its role as a recognized DAMP. This is similar to effects we have seen in other model systems (e.g. sepsis) where hepatocyte-derived HMGB1 is the main source of HMGB1 and drives inflammatory responses and also can induce inflammatory cell death (Deng et al. 2018). In addition, for the minor differences seen between our in vivo and in vitro experiments, we accept that PA treatment in a more acute setting in vitro in isolated hepatocytes does not necessarily recapitulate the in vivo findings.

## Conclusion

We demonstrate a novel protective role for intracellular hepatocyte HMGB1 in preventing HFD-mediated liver injury, in part through regulation of ER stress and fatty acid (FA) β-oxidation. Modulation of these pathways may therefore represent attractive future pharmacological targets to arrest NAFLD progression at an early stage. Whether the differences observed in HC-HMGB1^−/−^ and WT mouse strains are exclusively attributed to multiple differences in genetic background or whether the constitutive adaption of HMGB1^−/−^ hepatocytes plays a specific role in maintenance of liver ER homeostasis and lipid metabolism in this pathological state remains to be fully investigated. Further studies should be warranted to explore the possible HC-HMGB1-stablizingtherapeutic strategies for preventing the hepatic fat accumulation in early cases of NAFLD by way of regulating ER stress and FA β-oxidation.

## Supplementary information

**Additional file 1: Table S1.** Composition of rodent special diets. **Table S2.** Primer sequence used for RT-PCR.

**Additional file 2: Figure S1.** Food intake and energy expenditure in WT and HC-HMGB1^−/−^ mice up to 16 weeks after LFD or HFD feeding. **A:** Food intake levels. **B:** Energy expenditure (VO_2_). *P < 0.05 between WT LFD and KO LFD, or WT HFD and KO HFD groups; NS, not significant.

**Additional file 3: Figure S2.** **A:** Serum ALT and **B:** serum IL-6 levels WT and HC-HMGB1^−/−^ (KO) mice up to 16 weeks after LFD or HFD feeding. N = 8 for each group; data show mean ± SEM. *P < 0.05 between WT LFD and KO LFD, or WT HFD and KO HFD groups; NS, not significant.

## Data Availability

All supporting data including original/full length Western blots and original data are available upon request.

## Abbreviations

NAFLD: Nonalcoholic fatty liver disease
NASH: Non-alcoholic steatohepatitis
HMGB1: High-mobility group box 1
DAMP: Damage-associated molecular pattern
HC-HMGB1: Hepatocyte-specific HMGB1
ER: Endoplasmic reticulum
WT: Wild-type
HFD: High-fat diet
LFD: Low-fat diet
ALT: Alanine amino-transferase
IL: Interleukin
ELISA: Enzyme-linked immunosorbent assay
H&E: Hematoxylin and eosin
PA: Palmitic acid
OCR: Oxygen consumption rate
FCCP: Fluoro-carbonyl cyanide phenylhydrazone
TM: Tunicamycin
PBA: 4-Phenylbutyric acid
CPT-1α: Carnitine-palmitoyltransferase 1α
MCAD: Medium-chain acyl-CoA dehydrogenase
LCAD: Long-chain acyl-CoA dehydrogenase
VLCAD: Very-long chain acyl-CoA dehydrogenase
FAS: Fatty acid synthase
LPL: Lipoprotein lipase
SREBP-1: Sterol regulatory element-binding protein 1
PPAR-γ: Peroxisome proliferator-activated receptor gamma
COL1A1: Adipophilin, collagen type 1A1
COL1A2: Collagen type 1A2
TIMP: Tissue inhibitors of metalloproteinases
α-SMA: Alpha smooth muscle actin
SCD-1: Stearoyl-CoA desaturase-1
RT-PCR: Reverse-transcriptase polymerase chain reaction
p-Akt: Phospho-Akt
SEM: Standard error of mean
FFA: Free fatty acid
TLR: Toll-like receptors
RAGE: Receptor for advanced glycation end products
UPR: Unfolded protein response
rHMGB1: Recombinant HMGB1
SREBP: Sterol regulatory element-binding protein
DGAT: Diacylglycerol acyltransferase
VLDL: Very-low-density lipoprotein
FA: Fatty acid
