# Supplementary material for: Hepatocyte high-mobility group box 1 protects against steatosis and cellular stress during high fat diet feeding
Source: Mol Med. 2020 Nov 25;26:115. doi: 10.1186/s10020-020-00227-6 (PMC7687718; doi:10.1186/s10020-020-00227-6)
Supplement: Supplementary file 1 — Additional file 1: Table S1. Composition of rodent special diets. Table S2. Primer sequence used for RT-PCR. [file 10020_2020_227_MOESM1_ESM.docx]

**Additional Table 1. Composition of rodent special diets**

| **Special diets** | HFD | | LFD | |
| --- | --- | --- | --- | --- |
| **Products #** | D12451 | | D12450K | |
|  | gm% | kcal% | gm% | kcal% |
| Protein | 24 | 20 | 19.2 | 20 |
| Carbohydrate | 41 | 35 | 67.3 | 70 |
| Fat | 24 | 45 | 4.3 | 10 |
| Total |  | 100 |  | 100 |
| kcal/g | 4.73 |  | 3.85 |  |

### Additional Table 2. Primer sequence used for RT-PCR

| **Gene** | **Primer orientation** | **Sequence** |
| --- | --- | --- |
| CPT-1α | forward | 5′-GCTGCTTCCCCTCACAAGTTCC-3′ |
|  | reverse | 5′-GCTTTGGCTGCCTGTGTCAGTATGC-3′ |
| MCAD | forward | 5′-CAACACTCGAAAGCGGCTCA-3′ |
|  | reverse | 5′-ACTTGCGGGCAGTTGCTTG-3′ |
| LCAD | forward | 5′-TCACCACACAGAATGGGAGA-3′ |
|  | reverse | 5′-TTTCTCTGCGATGTTGATGC-3′ |
| VLCAD | forward | 5′-GAATGACCCTGCCAAGAACGA-3′ |
|  | reverse | 5′-ATGCCCACAATCTCTGCCAAG-3′ |
| FAS | forward | 5′-GCTGCGGAAACTTCAGGAAAT-3′ |
|  | reverse | 5′-AGAGACGTGTCACTCCTGGACTT-3′ |
| PPAR-γ | forward | 5′-TGCAGCTCAAGCTGAATCAC-3′ |
|  | reverse | 5′-ACGTGCTCTGTGACGATCTG-3′ |
| SCD-1 | forward | 5′-TGCCCCTGCGGATCTT-3′ |
|  | reverse | 5′-GCCCATTCGTACACGTCATT-3′ |
| Adipophilin | forward | 5′-GCACTGGCAAGTTCTACTGCAACA-3′ |
|  | reverse | 5′-AGAGAACGGCCTTGTCCTTCTTGA-3′ |
| LPL | forward | 5′- GCCCAGCAACATTATCCAGT-3′ |
|  | reverse | 5′- AGCCCTTTCTCAAAGGCTTC-3′ |
| SREBP1 | forward | 5′-TGACCCGGCTATTCCGTGA-3′ |
|  | reverse | 5′-CTGGGCTGAGCAATACAGTTC-3′ |
| β-actin | forward | 5′-CAAGATCATTGCTCCTCCTG-3′ |
|  | reverse | 5′-TCATCGTACTCCTGCTTGCT-3′ |
