# Supplementary figures and images for: Hepatocyte high-mobility group box 1 protects against steatosis and cellular stress during high fat diet feeding
Source: Mol Med. 2020 Nov 25;26:115. doi: 10.1186/s10020-020-00227-6 (PMC7687718; doi:10.1186/s10020-020-00227-6)

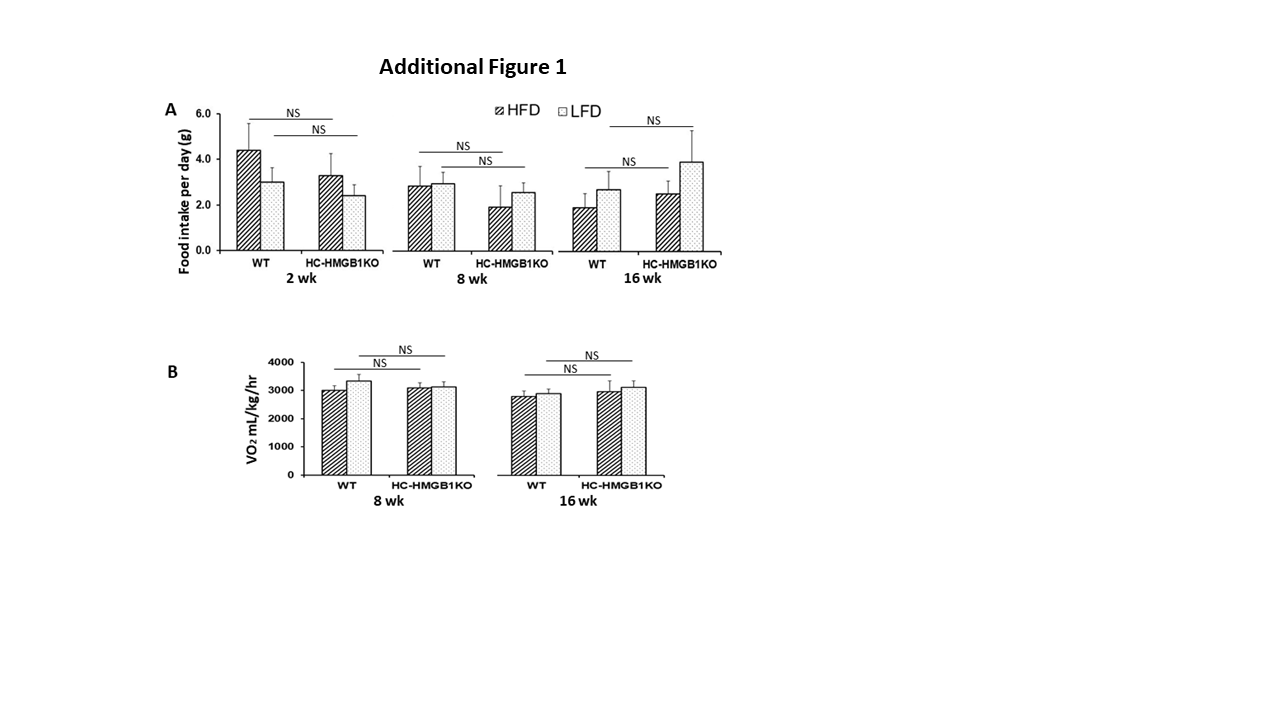

Supplement: Supplementary file 2 — Additional file 2: Figure S1. Food intake and energy expenditure in WT and HC-HMGB1−/− mice up to 16 weeks after LFD or HFD feeding. A: Food intake levels. B: Energy expenditure (VO2). *P < 0.05 between WT LFD and KO LFD, or WT HFD and KO HFD groups; NS, not significant. [file 10020_2020_227_MOESM2_ESM.tif]

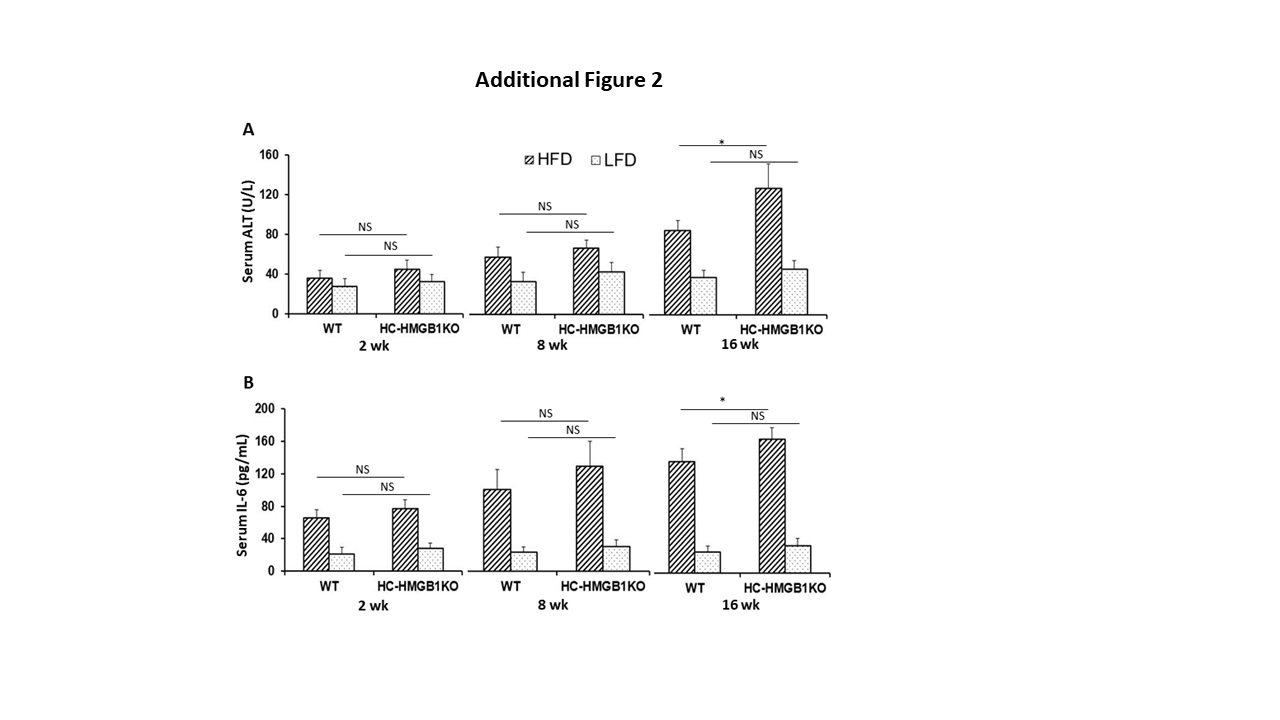

Supplement: Supplementary file 3 — Additional file 3: Figure S2. A: Serum ALT and B: serum IL-6 levels WT and HC-HMGB1−/− (KO) mice up to 16 weeks after LFD or HFD feeding. N = 8 for each group; data show mean ± SEM. *P < 0.05 between WT LFD and KO LFD, or WT HFD and KO HFD groups; NS, not significant. [file 10020_2020_227_MOESM3_ESM.tif]
